# The correlation between physical activity and psychological resilience in young students: a systematic review and meta-analysis

**DOI:** 10.3389/fpsyg.2025.1557347

**Published:** 2025-04-29

**Authors:** Wentao Qiu, Chao Huang, Haibin Xiao, Yuyang Nie, Wenxue Ma, Fangbing Zhou, Cong Liu

**Affiliations:** ^1^College of Physical Education and Sports, Beijing Normal University, Beijing, China; ^2^College of Education for the Future, Beijing Normal University, Zhuhai, China; ^3^College of Physical Education, Shangrao Normal University, Shangrao, China

**Keywords:** young students, physical activity, psychological resilience, association, influencing factors

## Abstract

**Objective:**

Against the backdrop of increasing social stress and a faster pace of life, psychological resilience as a key psychological attribute has become increasingly prominent. Physical activity is also a significant factor influencing the psychological resilience of young students. This study aims to systematically review the research progress on the relationship between physical activity and psychological resilience among young students. It analyzes the correlation between the two and the influencing factors, and explores the mechanisms by which physical activity among young students contributes to psychological resilience. The objective is to motivate young students to engage more actively in physical activities, thereby enhancing their psychological resilience.

**Methods:**

Following the Preferred Reporting Items for Systematic Reviews and Meta-Analyses (PRISMA) statement, an extensive literature search was conducted across six electronic databases: Web of Science, PubMed, ProQuest, Scopus, ScienceDirect, and EBSCOhost. The search spanned from January 1, 2000, to November 20, 2024. The selected studies were subjected to rigorous quality evaluation, and relevant methodological and outcome data were extracted using a standardized data extraction form. Subsequently, meta-analysis of the included studies was performed using Stata 18 software, including heterogeneity testing and assessment of publication bias.

**Results:**

After literature screening, a total of 21 studies were included, exploring the correlation between physical activity and psychological resilience. These studies employed Pearson correlation analysis (14 studies), multiple regression analysis (13 studies), and structural equation modeling (SEM) (eight studies). Using a random-effects model to combine effect sizes, the average correlation coefficient was 0.249 (*p* < 0.001), the average standardized coefficient for the promotion of psychological resilience by physical activity was 0.195 (*p* < 0.001), and the average path coefficient for the enhancement of psychological resilience through physical activity was 0.205 (*p* < 0.001).

**Conclusion:**

This study found a significant positive correlation between physical activity and psychological resilience among young students, with physical activity exerting a positive impact on psychological resilience. Similarly, psychological resilience plays a promotional role in the participation of young students in physical activities.

## Introduction

Adolescence, a pivotal period in one's life, serves as a catalyst for personal growth and is a crucial stage for the development of healthy psychological needs. During this time, learning to balance new demands is particularly critical. Family, school, and societal support that is comprehensible, perceptible, and acceptable to students can enhance their engagement in physical activities, thereby improving their mental health (Li et al., 2023). In 2023, China issued the “Comprehensive Strengthening and Improvement of Student Mental Health Work in the New Era Action Plan (2023–2025)” (hereinafter referred to as the “Action Plan”), which systematically advances mental health initiatives from eight dimensions with an emphasis on the “Five-Education” approach. The plan focuses on the key elements, areas, and links of student mental health, aiming to address deficiencies and strengthen weak points. The implementation of the action plan has exerted a significant and far-reaching impact on the comprehensive development of students' mental health.

Mental health issues among young students have increasingly become a focus of research. Academic pressures, interpersonal relationship challenges, and self-cognition problems they face can lead to negative emotions such as anxiety, depression, and low self-esteem, which not only affect academic performance and daily life but may also adversely impact their long-term development (Karyotaki et al., 2020; Yousif et al., 2022). Studies indicate that over 60% of students face at least one mental health issue (Lipson et al., 2022), and the prevalence of depression among Chinese university students is as high as 28.4% (Gao et al., 2020), potentially leading to negative behaviors including suicide and a decline in academic performance (Kalisch et al., 2017). Physical activity, an important factor influencing mental health, has a positive effect on increasing physical energy expenditure (Bull et al., 2020). Physical exercise, defined as bodily activity with a certain intensity, frequency, and duration, aims to enhance physical health (Liu, 2020). University students lacking in physical activity may suffer from skeletal and functional health impairments, as well as obesity, decreased vision, and psychological issues (Brown et al., 2024). Research by Carless and Douglas (2008) demonstrates that regular physical activity can effectively combat obesity, chronic diseases, and promote overall health, while also playing a positive role in personal identity formation and the prevention of mental disorders. Malm et al. (2019) found that physical activity can assist adolescents in overcoming negative emotions such as anxiety, depression, and stress, while fostering a positive psychological state. Studies in rehabilitation medicine and exercise science also confirm the positive effects of physical exercise on depression (Mota-Pereira et al., 2011; Schuch et al., 2011). Levante et al. (2024) found that high-frequency physical activity was associated with a positive psychological state, including optimism, self-actualization, and satisfaction in social interactions. Lin and Gao (2023) research revealed that physical exercise interventions positively contribute to alleviating anxiety symptoms among college students, with aerobic exercise identified as the optimal mode of exercise. Despite the recognized importance of physical exercise, participation among young students is low, with a declining trend in the proportion of students engaging in physical exercise (Nyberg et al., 2020; San Román-Mata et al., 2020). Most university students do not meet the recommended activity levels (European Opinion Research Group, 2002), and a study involving 15 European countries also revealed insufficient levels of physical activity participation (Ruiz et al., 2011). Individuals who reduce their physical activity are more likely to adopt unhealthy lifestyles and face psychological problems (Brunet et al., 2013). According to the World Health Organization (WHO) guidelines, adults should engage in at least 150–300 min of moderate to vigorous physical activity per week (Bull et al., 2020). Therefore, researching methods to increase the participation in physical activity and improve mental health levels among young students has become a critical area of academic study.

Mental health is a multidimensional concept involving emotions, cognition, behavior, and social interaction, which is central to overall wellbeing (Chaplin, 2015; Zhang, Y., 2022). Psychological resilience, as a significant psychological factor promoting physical activity participation among young students, has garnered widespread attention (Yu and Ye, 2023). Although limited research has explored this relationship, the association between resilience and goals, emotions, and cognition has been investigated (Chaplin, 2015; Zhang, Y., 2022). Studies that integrate psychological resilience with health behaviors are scarce (Crust and Keegan, 2010; Gerber et al., 2013), yet the concept of resilience holds considerable value in the field of sports psychology for understanding the relationship between physical activity and mental health (Crust and Keegan, 2010; Gerber et al., 2013). Psychological resilience is a commonly used term in applied sports psychology (Jones et al., 2002) and is widely regarded as an individual's adaptive capacity to effectively cope with life stressors and setbacks (Chen and Jiang, 2019). It not only reflects an individual's ability to deal with related stress and anxiety but also includes the capacity to maintain determination, confidence, and control under pressure (Crust, 2008; Mack and Ragan, 2008). Psychological resilience is not a fixed trait but a dynamic concept that changes over time (Rutter, 2023). Fleshner (2005) proposed that regular participation in moderate physical activity can enhance psychological resilience and alleviate stress, whereas a lack of such activity may lead to increased body weight in adolescents, potentially resulting in overweight or obesity (Micheletti Cremasco et al., 2021). This study, through a systematic review, explores the association between physical activity and psychological resilience in young students, aiming to reveal the intrinsic mechanisms of their physical activity participation from a psychological motivational perspective. Additionally, it aims to provide a scientific basis for the development of effective behavioral intervention strategies to enhance physical activity levels and improve overall health outcomes.

However, there is a scarcity of review studies on the impact of physical activity on the psychological resilience of adolescent students, with existing research primarily focusing on college student populations. For instance, Seçer and Yildizhan (2020) found a positive correlation between physical activity levels and psychological resilience in college students, and Li and Guo (2023) also indicated that physical activity significantly predicts psychological resilience and social adaptation among college students. This research bias may overlook the importance of strategies to promote physical activity among adolescents. Given the physiological and psychological developmental differences between adolescents and adults, the formation and mechanisms of action of their psychological resilience may differ from those of adults. Therefore, studying the psychological resilience of adolescent students is crucial for understanding their specific needs and challenges in physical activity.

Additionally, previous research has fallen short in exploring the relationship between psychological resilience and the general healthy population, with a primary focus on special groups. For instance, Matzka et al. (2016) found a significant positive correlation between the psychological resilience of cancer patients and their level of physical activity. Currently, there is a paucity of review studies on psychological resilience, which may overlook its unique role in promoting physical activity. Mental health involves a general sense of confidence in facing challenges, whereas psychological resilience more specifically pertains to the level of confidence individuals have in coping with challenges. Since physical activity directly impacts psychological resilience, research in this area is crucial for developing intervention strategies targeting the psychological resilience of young students.

In summary, this study aims to systematically review the association between exercise-related psychological resilience and physical activity among young students, organize and interpret relevant research data. The goal is to provide theoretical support for enhancing the psychological resilience and improving the quality of life of young students, as well as to offer solutions to address the psychological issues currently faced by this demographic.

## Methods

### Literature search strategy

A structured electronic literature search was conducted in accordance with the standards set forth in the Preferred Reporting Items for Systematic Reviews and Meta-Analyses (PRISMA) statement. The search was performed by the first author on October 20, 2024, and included six electronic databases (Web of Science, PubMed, ProQuest, Scopus, SAGE Journals, and EBSCO). The search strategy was as follows: [Title/Abstract] = (“physical activity” OR “physical exercise” OR “physical training” OR “fitness activity”) AND [Title/Abstract] = (“mental toughness” OR “psychological resilience” OR “psychological capacity”) AND [Title/Abstract] = (“young students” OR “student” OR “youth”). Only peer-reviewed literature published in English or Chinese was considered for inclusion. The search covered a time span from January 1, 2000, to November 20, 2024.

### Inclusion and exclusion criteria

Inclusion criteria: ① Participants aged between 12 and 25 years; ② Studies that included an assessment of psychological resilience; ③ Studies that included an assessment of physical activity; ④ Quantitative analysis of the association between psychological resilience and physical activity; ⑤ Cross-sectional, longitudinal, or long-term research designs; ⑥ Publications in English.

Exclusion criteria: ① Studies focused on special populations, such as those with cardiovascular disease, diabetes, etc.; ② Literature not published in peer-reviewed journals; ③ Studies with a sample size of < 100 participants; ④ Studies that did not provide data on the association between psychological resilience and physical activity; ⑤ Review or regression analysis literature.

After de-duplication of the retrieved literature, two researchers independently screened the studies based on the inclusion and exclusion criteria. A preliminary screening of titles and abstracts was conducted to identify potentially relevant literature for full-text review. Additionally, while including studies confirmed through full-text review, we also meticulously examined the reference lists of the retrieved full-text articles and other systematic reviews to ensure that no eligible additional studies were missed. Finally, the two researchers cross-checked to determine the studies to be included. In case of discrepancies in the screening results, a third researcher was consulted to make the final decision.

### Data extraction

Data were extracted independently by two authors based on the inclusion criteria, with discrepancies resolved through discussion. The following information was extracted: (1) Author and year of publication; (2) Study location; (3) Study type; (4) Methods of assessing psychological resilience; (5) Methods of assessing physical activity; (6) Statistical methods; (7) Association indicators (correlation coefficients, standardized coefficients); (8) Study findings.

### Quality assessment of the literature

Literature quality assessment was conducted using the criteria from the Quality Assessment Tool for Primary Research Papers from Different Areas (QualSyst) (Kmet et al., 2004). QualSyst is an instrument comprising 14 items that allows for methodological and bias assessment in both quantitative and qualitative studies with varying research designs. Due to the observational design of the studies included in this review, items 5 (random allocation), 6 (researcher blinding), and 7 (subject blinding) were omitted from the QualSyst. Each item on the QualSyst was scored from 0 to 2, indicating whether the study met a criterion (0 = no, 1 = partially, 2 = yes). All scores were summed to create a total score. The total score was then converted into a percentage score (i.e., the study's total score divided by 22), with ratings of “excellent” (>80%), “good” (70–79%), “sufficient” (55–69%), and “low” (< 55%) (Selzler et al., 2020; Fang et al., 2021). Two researchers independently assessed the quality, with discrepancies resolved through consultation until a consensus was reached.

### Statistical analysis

Most of the studies included provided data on the association between psychological resilience indicators and physical activity in young students, as well as the sample size of the studies. For longitudinal studies included, the association data at baseline were selected for analysis; for studies that used multiple instruments for assessment, the association data were combined using meta-analysis. The transformed data were subjected to meta-analysis using Stata 18 software. Based on the results of the heterogeneity analysis, a fixed-effect model was adopted for the included literature when *I*^2^ was < 50% and *P* was >0.05, or a random-effects model when *I*^2^ was ≥ 50% or *P* was < 0.05. The level of heterogeneity was represented by the *I*^2^ index, categorized as low (*I*^2^ ≤ 25%), moderate (25% < *I*^2^ ≤ 50%), and high (*I*^2^ > 50%) (Liu et al., 2023; Higgins et al., 2003). Subgroup analysis was conducted in the presence of significant heterogeneity. To test for publication bias, funnel plots were calculated and Egger's test was performed, with the results of the analysis presented in forest plots.

## Results

### Study selection process

**Figure 1:**
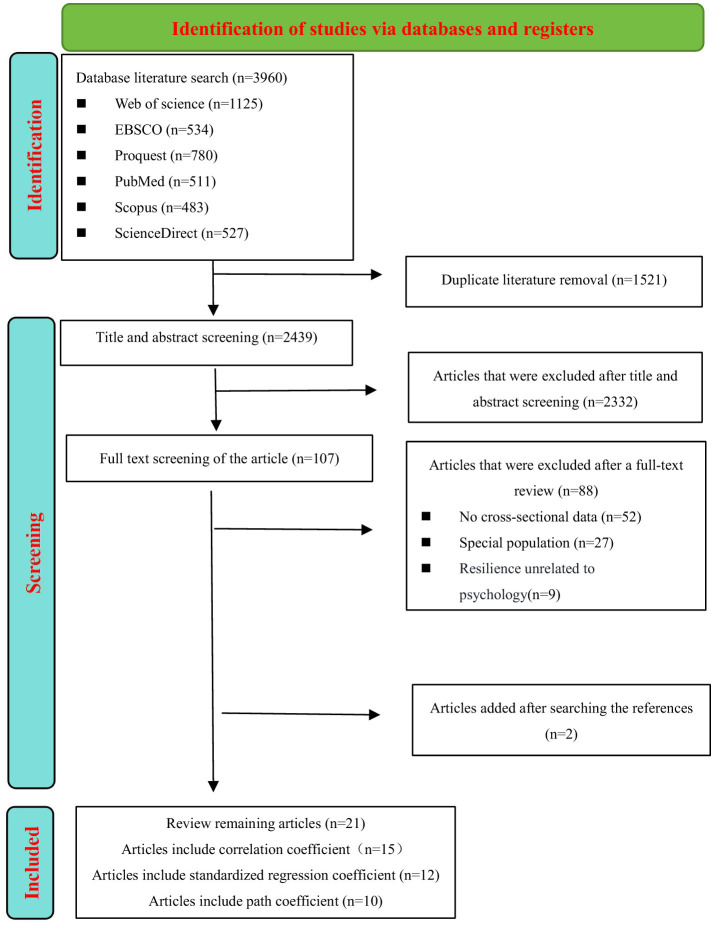
Flow chart of literature screening.

Figure 1 illustrates the study screening process and the reasons for excluding literature. The initial database search retrieved 3,960 potentially relevant articles. After duplicates were removed, 2,439 unique articles entered the title and abstract screening phase, of which 2,332 were excluded. Subsequently, the full texts of 107 articles were reviewed in detail against the inclusion criteria. During this process, 88 articles were excluded for the following reasons: 52 lacked original data, 27 focused on special populations (e.g., patients with diseases, individuals with movement disorders), and nine did not address psychological resilience. Ultimately, a total of 21 articles met the inclusion criteria, spanning from January 2000 to November 2024.

### Basic characteristics of the included studies

Table 1 summarizes the basic information of the 21 studies included in this review. All studies were published after the year 2000, comprising 20 cross-sectional studies and 1 longitudinal study, with all providing cross-sectional data. The study locations spanned multiple countries including the United States and China, with the number of participants ranging from 124 to 2,375 individuals. Among the included studies, 14 conducted correlation analyses and reported correlation coefficients (*r*), 13 employed multiple regression analyses and provided standardized coefficients (β) along with standard errors (SE), and eight utilized structural equation modeling and reported consistent path coefficients (γ). These studies also presented estimated effect sizes with their corresponding 95% confidence intervals. Detailed information is provided in Table 1.

**Table 1:** Summary of the included literature.

**Number**	**Author, Year**	**Study design**	**Country**	**Sample size**	**Age (years) mean (SD) [range]**	**Percentage girls (%)**	**Instruments used (PA)**	**Instruments used (PR)**	**Analysis**	**Associationindicators**	**Conclusion**	**References**
1	Yongbin Li, 2023	CS	CN	1,622	18–23 (21.18)	44.94%	PARS-3	BRS	Correlation analysis Regression analysis Mediation effect analysis	*r* = 0.215, *p* < 0.01 β = 0.2145	Physical activity can significantly positively predict the psychological resilience of college students.	Li and Guo, 2023
2	Na Li, 2024	CS	CN	1,106	(15.7)	39.2%	PARS-3	CD-RISC	Pearson correlation analysis Mediation effect analysis SEM	*r* = 0.194 β = 0.119 β = 0.119	Exercise behavior is positively correlated with psychological resilience and the need for belonging.	Li et al., 2024
3	Emrah Seçer, 2020	QR	TR	1,734	17–22	58.2%	IPAQ	PRS	Correlation analysis Regression analysis	*r* = 0.17, *p* < 0.01 β = 0.176	There is a positive low-level relationship between physical activity levels and levels of psychological resilience.	Seçer and Yildizhan, 2020
4	Lingling Guo, 2023	LS	CN	818	12–17	45.2%	PARS-C	ARRS	Partial correlation analysis Cross-lagged panel model	ST1 and RT1 (*r* = 0.722) ST2 and RT2 (*r* = 0.837)	It has been confirmed that there is a significant positive correlation between physical activity and psychological resilience.	Guo and Liang, 2023
5	Mahta	CS	IR	355	21.2		HPA	MTQ48	Pearson correlation analysis	*r* = 0.216, *p* ≤ 0.01	A positive correlation exists between physical activity and psychological resilience among the college student population.	Eskandarnejad, 2015
6	Ming Liu, 2024	CS	CN	1,380	17–25	44.0%	PARS-3	CD-RISC	Correlation analysis Mediation effect analysis Regression analysis	*r* = 0.270, *p* < 0.001 β = 0.255, *p* < 0.001	Physical activity is positively correlated with psychological resilience and proactive coping styles, and negatively correlated with negative emotions.	Liu et al., 2024
7	Guoqing Cui, 2022	CS	CN	1,048	19–23 (20.12)	48.3%	PARS	ARRS	Bivariate Pearson correlation analysis Regression analysis Moderation effect analysis	β = 0.387, *P* < 0.001	Physical exercise can effectively alleviate negative emotions in college students, with self-efficacy and psychological resilience playing a mediating role, and family support can moderate this relationship.	Cui and Zhang, 2022
8	Emily R. Dunston, 2020	CS	US	244	(21.1)	67.6%	IPAQ-S	CD-RISC	Correlation analysis Linear regression analysis Mediation analysis	*r* = 0.16, *p* = 0.01 β = 0.17, *p* = 0.01 β = 0.15, *p* = 0.02	Intense exercise is associated with higher resilience and perseverance.	Dunston et al., 2022
9	Markus Gerber, 2012	CS	CH	284	(18.3)	64.8%	IPAQ	MTQ48	Pearson's product-moment correlation		Exercise and physical activity are positively correlated with psychological resilience.	Gerber et al., 2012
10	Zitong Zhao, 2022	CS	CN	257	18–25	71.6%	PARS-3	CD-RISC	SEM	β = 0.195, *p* < 0.05	Physical exercise can first enhance psychological resilience, thereby alleviating perceived stress, and ultimately reducing smartphone addiction.	Zhao et al., 2022
11	Peggy Cheung, 2019	CS	CN	1,209	12–21 (14.85)	39.8%	PAQ-C	MTI	LPAs	*r* = 0.28, *p* < 0.001	There is a significant positive correlation between physical activity and psychological resilience.	Cheung and Li, 2019
12	Serge Brand, 2016	CS	CH	1,361	11–16 (13.37)	16.2%	IPAQ	MTQ18	ANOVA		During early to mid-adolescence, higher levels of physical activity are associated with improved sleep and psychological functioning, including increased curiosity, exploratory behavior, and psychological resilience.	Brand et al., 2017
13	Abdurrahman Demi*r*, 2020	CS	TR	360	(20.73)	56.7%	IPAQ	SPRS	Pearson correlation test Mann-Whitney *U*-test	*U* = 7,426.000, *p* = 0.003	Regular exercise has a positive impact on the psychological resilience and anxiety levels of college students. Those who engage in moderate physical activity tend to have higher scores in psychological resilience.	Demir and Barut, 2020
14	Jane Jie Yu, 2023	CS	CN	352	(20.8)	85.9%	IPAQ-SF	CD-RISC-25	Mann-Whitney *U*-test GLMs	OR = 1.023 (95% CI = 1.002, 1.045)	Psychological resilience is an important predictor for college students to meet the recommended levels of physical activity, particularly for moderate and high-intensity activities.	Yu and Ye, 2023
15	Mengmeng Yang, 2024	CS	CN	1,350	(20.43)	44.8%	PARS	SRS	Correlation analysis Regression analysis Moderation effect analysis	*r* = 0.414, *p* < 0.01 β = 0.574, *p* < 0.01	There is a significant positive correlation between physical activity, cognitive reappraisal, psychological resilience, and subjective wellbeing.	Yang et al., 2024
16	Huiru Lin, 2024	CS	CN	542	13–18	55.4%	PARS-3	RSCA	Correlation analysis Moderation effect analysis Hierarchical regression analysis	*r* = 0.24, *p* < 0.01 β = 0.11, *p* < 0.01	Physical activity is significantly positively correlated with self-esteem, psychological resilience, and interpersonal adaptability.	Lin et al., 2024
17	Shanshan Xu, 2021	CS	CN	2,375	(20.25)	53.26%	IPAQ-SF	CD-RISC	Correlation analysis Regression analysis Moderation effect analysis	*r* = 0.159, *p* < 0.01 β = 0.151, *p* < 0.001 Direct effect = 0.051	This study indicates that physical activity is significantly positively correlated with the resilience of college students.	Xu et al., 2021
18	Xinbo Wu and Junwen Liang, 2024	CS	CN	1,488	12–16 (13.59)	48.3%	PARS-3	RSA	Correlation analysis Moderation effect analysis	*r* = 0.581, *p* < 0.01 Direct effect β = 0.52	There is a significant positive correlation between physical activity and psychological resilience.	Wu et al., 2024
19	Inger E. O. Moljord, 2014	CS	NO	1,110	13–18	49%	SIM	READ	Correlation analysis Multilevel linear regression analysis Interaction effect analysis	*r* = 0.115	Psychological resilience is a good predictor of depressive symptoms, whereas the impact of physical activity is relatively minor.	Moljord et al., 2014
20	Zhihao Zhang, 2022	CS	CN	1,117	(18.90)	50.4%	IPAQ-SF	CD-RISC	Correlation analysis Stepwise mediation model analysis	*r* = 0.098, *p* < 0.01	Physical activity can enhance exercise tolerance and psychological resilience, thereby reducing negative emotional states and having a positive impact on the mental health of college students.	Zhang, Z. et al., 2022
21	Haixiao	CS	CN	124	Freshman - Senior (or Fourth Year)	53.23%	PARS-3	MTQ-10	Multiple linear regression analysis Path analysis	*r* = 0.206 β = 0.135 β = 0.265	College students who frequently participate in physical activities have higher psychological resilience than those who do not engage in physical activities.	Xu, 2022

C, Cross sectional study; LS, Longitudinal study; IPAQ, International Physical Activity Questionnaire; PARS, Physical Activity Rating Scale; PAQ-C, Physical Activity Questionnaire for Older Children; HPA, Habitual physical activity questionnaire; SIM, Single-item measurement; BRS, Brief Resilience Scale; MTQ, Mental Toughness Questionnaire; CD-RISC, Connor-Davidson Resilience Scale; RSCA, Resilience Scale for Chinese Adolescents; SPRS, Short Psychopathy Rating Scale; PRS, Psychological Resilience Scale; MTI, Mental Toughness Index; ARRS, Adolescent Resilience Rating Scale; SPRS, Short Psychological Resilience Scale; SRS, sport resilience scale; RSA, resilience Scale for Adolescents; READ, self-reported Resilience Scale for Adolescents.

### Quality assessment of the included studies

The quality of the 21 included articles was assessed using the “Quality Assessment Tool for Primary Research Papers from Different Areas.” The scores of the 21 studies ranged from 68.2% to 100%, with an average score of 92.4%. Of these, 20 studies (95%) were rated as excellent quality, 1 study (5%) was rated as good quality, and none (0%) were rated as sufficient or poor quality, as shown in Table 2.

**Table 2:** Results of the quality assessment of the studies included in the systematic review (*N* = 21).

**Author and year**	**1. Research question**	**2. Study design**	**3. Subject and variable selection**	**4. Subjectcharacteristics**	**8. Exposuresand outcome**	**9. Samplesize**	**10. Analyticmethods**	**11. Estimateof variance**	**12. Confounding**	**13. Results in sufficient detail**	**14. Conclusions supporting results**	**Sums**	**Weights^†^**	**Rank^#^**
Yongbin Li, 2023	2	2	2	2	2	2	2	2	2	2	2	22	100.0%	Excellent
Na Li, 2024	2	1	2	2	2	2	2	2	2	2	2	21	95.5%	Excellent
Emrah Seçer,2020	2	2	2	2	2	2	2	2	2	2	2	22	100.0%	Excellent
Lingling Guo, 2023	2	2	2	2	2	2	2	2	2	2	2	22	100.0%	Excellent
Mahta Eskandarnejad, 2015	2	1	1	1	2	1	2	2	0	2	1	15	68.2%	Adequate
Ming Liu, 2024	2	2	2	2	2	2	2	1	2	2	2	21	95.5%	Excellent
Guoqing Cui, 2022	2	2	2	2	1	2	2	2	1	2	2	20	90.9%	Excellent
Emily R. Dunston, 2020	2	1	1	2	2	2	2	2	2	2	2	20	90.9%	Excellent
Markus Gerber, 2012	2	1	2	2	2	1	2	1	2	2	2	19	86.4%	Excellent
Zitong Zhao, 2022	2	2	2	2	2	2	2	1	2	2	2	21	95.5%	Excellent
Peggy Cheung, 2019	2	1	2	2	2	2	2	1	1	2	2	19	86.4%	Excellent
Serge Brand,2016	2	1	2	2	1	2	2	1	1	2	2	18	81.8%	Excellent
Abdurrahman Demir, 2020	2	2	2	2	2	1	2	1	1	2	2	19	86.4%	Excellent
Jane Jie Yu, 2023	2	1	2	2	1	2	2	2	1	2	2	19	86.4%	Excellent
Mengmeng Yang, 2024	2	2	2	2	2	2	2	2	2	2	2	22	100.0%	Excellent
Huiru Lin, 2024	2	2	2	2	2	2	2	2	2	2	2	22	100.0%	Excellent
Shanshan Xu, 2021	2	1	2	2	2	2	2	1	2	2	2	20	90.9%	Excellent
Xinbo Wu Junwen Liang, 2024	2	2	2	2	2	2	2	2	2	2	2	22	100.0%	Excellent
Inger E. O. Moljord, 2014	2	2	2	2	2	2	2	1	2	2	2	21	95.5%	Excellent
Zhihao Zhang, 2022	2	1	2	2	2	2	2	2	2	2	2	21	95.5%	Excellent
Haixiao Xu, 2022	2	2	2	2	2	2	2	1	2	2	2	21	95.5%	Excellent

Each item was scored depending on to what degree the criterion was met: yes = 2 points, partial = 1 point, no = 0. ^†^Study total sum score divided by the total possible score of 22.

^#^A percentage score of >80%, 70–80%, 55–69%, and < 55% was rated as “excellent,” “good,” “adequate”, and “low”, respectively.

### Assessment tools for physical activity among young students

The literature included in this study employed five tools to assess the frequency and energy expenditure of physical activity among young students. Of these, 10 studies used the Physical Activity Rating Scale (PARS) (Liang, 1994), a widely used scale to evaluate adolescent participation in physical activities, which quantifies exercise through three questions covering intensity, duration, and frequency. Eight studies utilized the International Physical Activity Questionnaire (IPAQ) (Craig et al., 2003). One study applied the Habitual Physical Activity Questionnaire (HPA) (Eskandarnejad, 2015), which not only collects basic information such as age and gender but also records the minutes of moderate to vigorous physical activity per week and assesses perceived exercise, encompassing domains of occupational, leisure-time physical exercise, and leisure and sports activities. Other studies used the Single-item Measurement (SIM) (Rangul et al., 2008) to evaluate the frequency of vigorous physical activity, as well as the Physical Activity Questionnaire for Older Children (PAQ-C) (Janz et al., 2008) targeting physical activity levels in children and adolescents aged 8–14 years. These tools provide a multidimensional perspective for assessing physical activity in young students, including activity frequency and the quantification of energy expenditure based on MET values.

### Assessment tool for the psychological resilience of young students

Among the included studies, six tools were employed to assess the psychological resilience of young students. Seven studies utilized the Connor-Davidson Resilience Scale (CD-RISC) (Connor and Davidson, 2003), which comprises three dimensions—resilience, strength, and optimism—with a total of 25 items. Four studies used the Mental Toughness Questionnaire (MTQ) (Clough et al., 2002), covering four dimensions: challenge, control, commitment, and confidence. The Brief Resilience Scale (BRS) (Chen et al., 2020) and the Resilience Scale for Chinese Adolescents (RSCA) (Hu and Gan, 2008) were each used in one study; the former consists of six items assessing the ability to bounce back from stress, while the latter is divided into five factors. The Psychological Resilience Scale (PRS) (Işik, 2016), the Adolescent Resilience Rating Scale (CYRM) (Liebenberg et al., 2012), and the Short Psychological Resilience Scale (SPRS) (Doǧan, 2015) were each used in one to two studies, containing 21, 28, and 6 items, respectively. Additionally, the Sport Resilience Scale (SRS) (Sheard et al., 2009), the Resilience Scale for Adolescents (RSA) (Hu and Gan, 2008), the Self-reported Resilience Scale for Adolescents (READ) (Hjemdal et al., 2006), and the Mental Toughness Index (MTI) (Gucciardi et al., 2015) were each employed in one study, assessing various dimensions and levels of psychological resilience. These scales offer a diverse range of methods for evaluating the psychological resilience of young students.

### Meta-analysis

**Figure 2:**
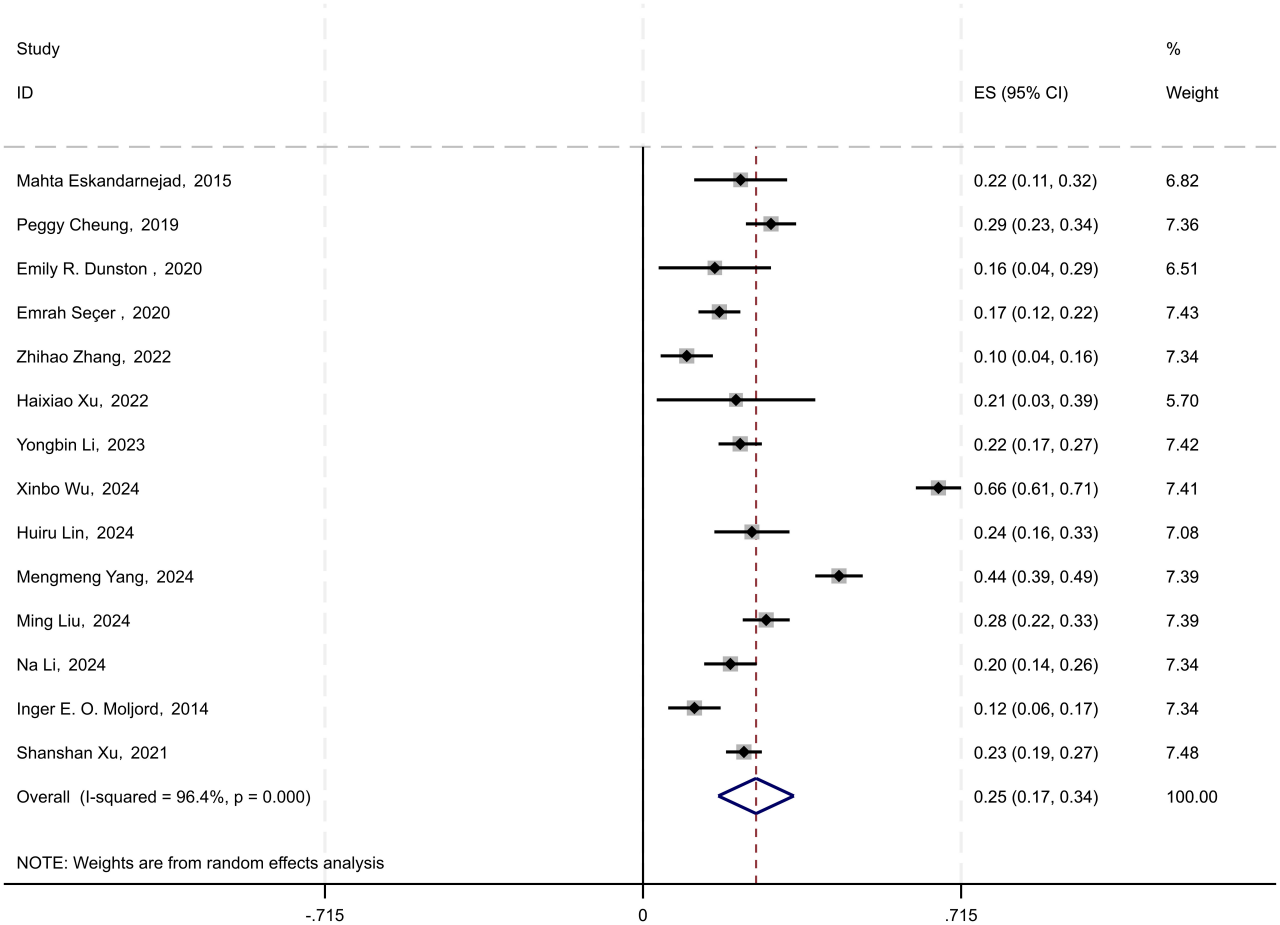
Forest plot of the relationship between physical activities and psychological resilience among young students. Each black square represents a separate study result. The diamond symbol represents the overall effect size of all studies.

In the 21 studies included, 14 provided data on the correlation between physical activityand psychological resilience in young students. To standardize the analysis, the Spearman correlation coefficient ρ was converted to the Pearson correlation coefficient *r* using the formula (*r* ≈ 6 sin(πρ/6)/π). Subsequently, by combining the Pearson coefficients with sample sizes, the data were transformed into Fisher's *Z*-scores, standard errors (SE), and 95%confidence intervals (CIs) (Borenstein et al., 2021; Hoare et al., 2016). A random-effects model was used to conduct a meta-analysis of the correlation coefficient *r* and its 95% CI. The results indicated a combined effect size of 0.254 (95% CI: 0.169–0.339), suggesting a significant overall effect across studies. The heterogeneity test yielded a chi-square value of 365.25 with 13 degrees of freedom, and a *p*-value < 0.0001, indicating substantial heterogeneity among the studies. The I-squared value of 96.4% implied that 96.4% of the variability in effect sizes could be attributed to between-study heterogeneity. Moreover, the *z*-value for the test of ES = 0 was 5.85, with a *p*-value < 0.0001, further substantiating the statistical significance of the findings (see Figure 2).

After converting the combined Fisher's *Z*-scores and their 95% CI back to the correlation coefficient *r*, the derived correlation coefficient was 0.249 (95% CI: 0.167–0.327). Consequently, the analysis revealed an average correlation coefficient of 0.249 (95% CI: 0.167–0.327) between physical activity and psychological resilience in young students.

**Figure 3:**
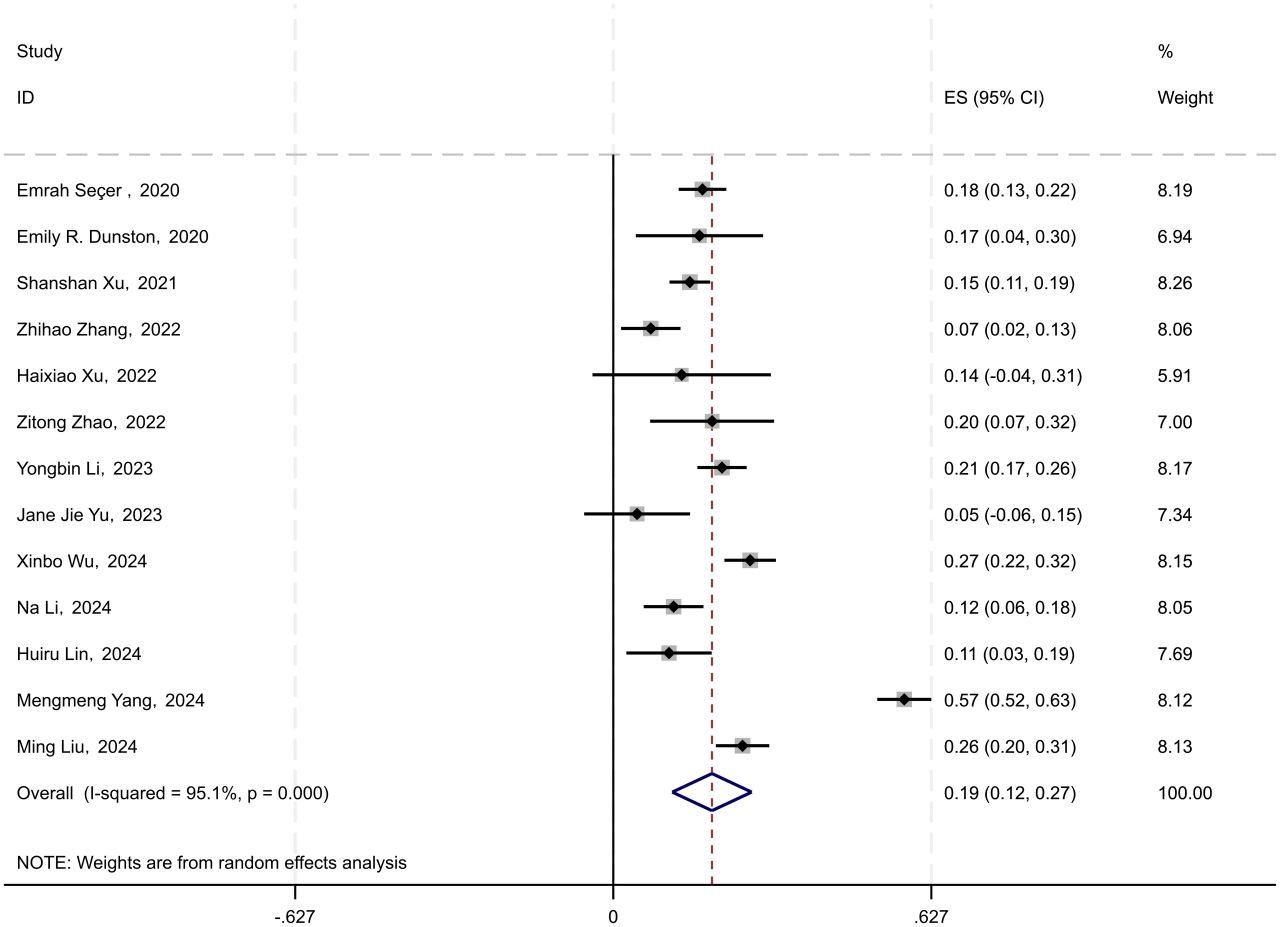
Forest plot of the impact of physical activity on psychological resilience in young students.

Among the 21 included studies, 13 employed multiple regression analysis and provided the effect sizes (standardized coefficients) for the impact of psychological resilience on physical activity among young students. The 95% confidence intervals were calculated using the formula (CI = β ± 1.96 × SE), based on the standardized coefficient β and the standard error SE. A random-effects model was used to conduct a meta-analysis of the standardized coefficients β and their 95% CI. The results revealed a combined effect size of 0.195 (95% CI: 0.116–0.273), indicating a significant overall effect across studies. The chi-square value for the heterogeneity test was 242.75 with 12 degrees of freedom, and the *p*-value was < 0.0001, suggesting significant heterogeneity among the studies. The *I*-squared value of 95.1% indicated that 95.1% of the variability in effect sizes could be attributed to heterogeneity between studies. Furthermore, the *z*-value for the test of ES = 0 was 4.87, with a *p*-value < 0.0001, further confirming the statistical significance of the findings (see Figure 3).

The comprehensive analysis results indicate that physical activity has a moderate positive impact on the psychological resilience of young students, with a standardized coefficient of 0.195. This implies that for every one standard deviation increase in physical activity levels, the psychological resilience of young students is expected to increase by an average of 0.195 standard deviation units.

**Figure 4:**
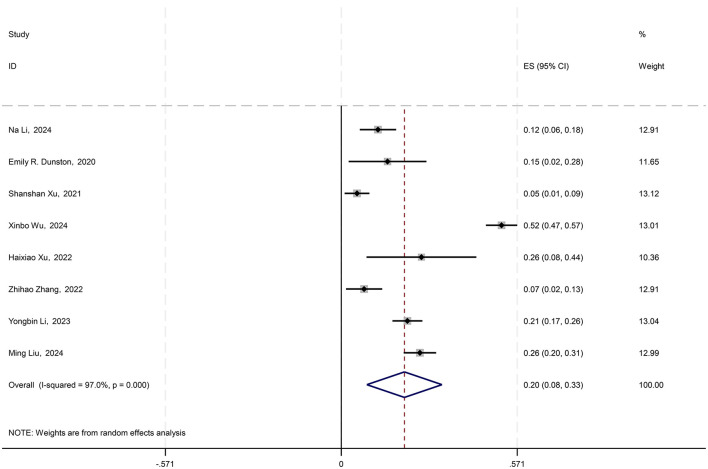
Forest plot of the impact of physical activity on psychological resilience in young students.

In the 21 studies included, eight employed structural equation modeling and provided effect sizes (path coefficients) for the impact of physical activity on the psychological resilience of young students. Using the path coefficient γ and sample size n, the standard error (SE) and 95% confidence interval (CI) were calculated with the formulas (SE = γ/√n, 95% CI = γ ± 1.96 × SE). A random-effects model was used to conduct a meta-analysis of the path coefficient γ and its 95% CI. The results indicated a combined effect size of 0.205 (95% CI: 0.083–0.327), suggesting a significant overall effect across studies. The chi-square value for the heterogeneity test was 235.17 with 7 degrees of freedom, and the *p*-value was < 0.0001, indicating substantial heterogeneity among the studies. The *I*-squared value of 97.0% suggested that 97.0% of the variability in effect sizes could be attributed to between-study heterogeneity. Additionally, the *z*-value for the test of ES = 0 was 3.29, with a *p*-value < 0.0001, further confirming the statistical significance of the findings (see Figure 4).

The comprehensive analysis revealed that physical activity among young students has a moderate positive impact on psychological resilience, with an average path coefficient γ of 0.205. This indicates that as the level of physical activity engaged in by young students increases, their psychological resilience also increases correspondingly, with an estimated enhancement of ~20.5% relative to the change in activity level.

### Subgroup analysis

**Figure 5:**
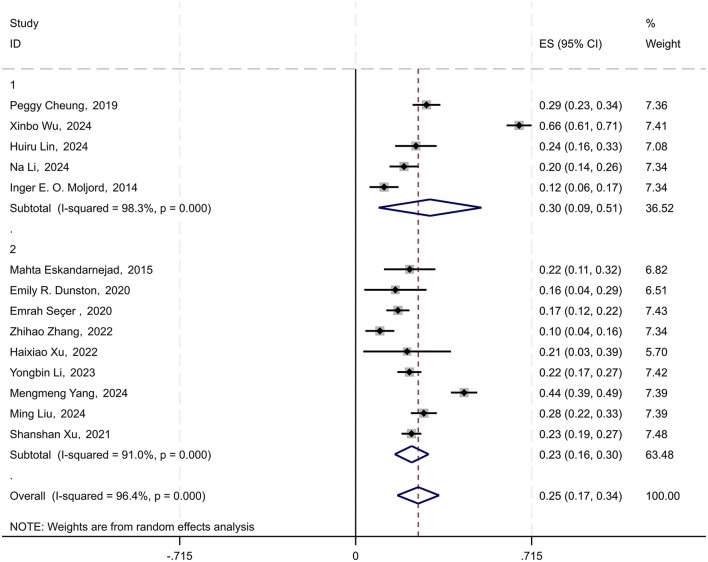
Forest plot of subgroup analysis. Each black square represents a separate study result. The diamond symbol represents the overall effect size of all studies.

This study also conducted a subgroup analysis based on age groups. Specifically, the data were divided into two subgroups: adolescents and college students. There were five studies related to adolescents and nine studies related to college students. The results of the subgroup analysis revealed a combined effect size of 0.302 (95% CI: 0.093–0.511) for the adolescent subgroup and 0.228 (95% CI: 0.159–0.296) for the college student subgroup. The *p*-values for both subgroups were < 0.001, indicating a significant effect across studies. The heterogeneity tests showed I-squared values of 98.3% for adolescents and 96.4% for college students, suggesting significant heterogeneity among studies (see Figure 5).

After converting the combined Fisher's *z*-scores and their 95% CIs back to correlation coefficients (*r*) and their 95% CIs, the correlation coefficient between physical activity and psychological resilience for the adolescent subgroup was *r* = 0.293 (95% CI: 0.093–0.471), and for the college student subgroup, it was *r* = 0.224 (95% CI: 0.158–0.288).

### Publication bias assessment

**Figure 6:**
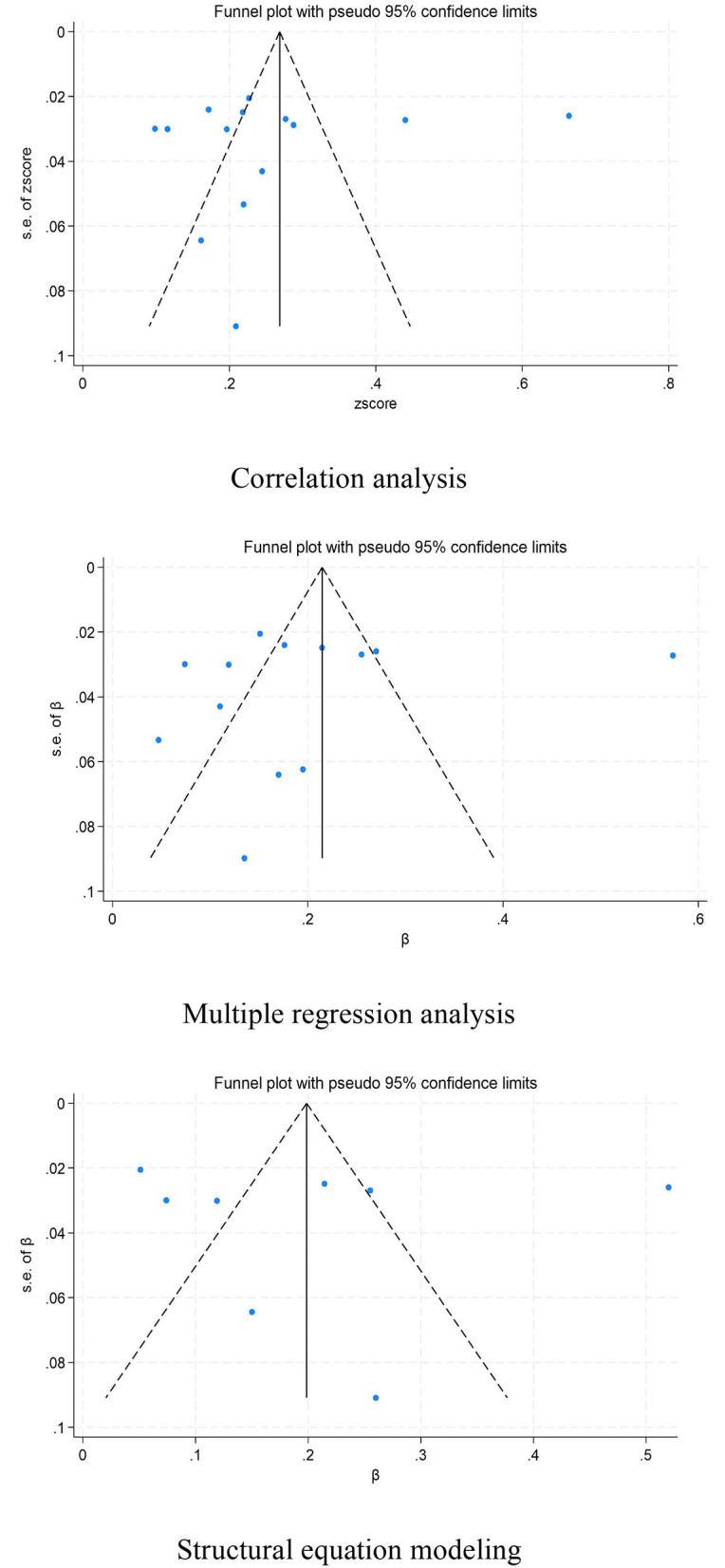
Funnel plot analyzing the relationship between physical activities and psychological resilience in young students.

A funnel plot and the Egger's test were utilized to examine publication bias in the included studies. The symmetry of the funnel plot (Figure 6) along with the *P*-values for the intercept coefficients from the Egger's test for different data types (0.686, 0.593, and 0.869) indicate the absence of publication bias in the statistical correlation data (*r*). Furthermore, there is no sufficient evidence to suggest the presence of small-study effects or publication bias in the standardized coefficients (β) and path coefficients (γ).

## Discussion

This study comprehensively and systematically reviews the relationship between physical activity and psychological resilience among young students, as well as the factors influencing this association. Through systematic database searches, we identified 21 research articles, encompassing both cross-sectional and longitudinal study designs, with an overall high quality of literature. To ensure the reliability of the findings in our meta-analysis, we included only studies with a sample size of 100 or more participants. This decision was based on the understanding that larger sample sizes enhance statistical power and the generalizability of the results. Moreover, studies with larger samples better represent the population, reduce the risk of biases and errors in data collection and analysis, and contribute to higher-quality research outcomes. This approach aligns with the prevalent standards and practices of large-sample research in the current field. The methodologies employed primarily included correlation analysis, multiple regression analysis, and structural equation modeling. We standardized similar correlation data and conducted a meta-analysis. The results revealed a significant statistical association between physical activity and psychological resilience in young students.

To further explore this association, we conducted a subgroup analysis of the correlation data between physical activity and psychological resilience, categorizing the participants into adolescents and college students. The findings indicated a significant positive correlation between physical activity and psychological resilience within both subgroups, suggesting the presence of this association across different age groups. However, the heterogeneity test among subgroups also highlighted differences between studies, which may be attributed to variations in psychological development characteristics, lifestyle, sample characteristics, study design, and measurement tools and methods between adolescents and college students. The greater heterogeneity observed in the adolescent subgroup may stem from the rapid psychological and physiological development during this stage, leading to significant individual differences that could affect the stability of research outcomes. Sample size also influences heterogeneity among studies; for instance, the smaller sample size in Xu (2022) may affect the representativeness of the sample and the heterogeneity of the results. Additionally, differences in measurement approaches can contribute to variability in heterogeneity among studies, such as the potential for assessment bias in the study by San Román-Mata et al. (2020), which relied on a self-administered questionnaire.

Moreover, we examined publication bias in the included literature. Neither the funnel plot nor the Egger's test revealed publication bias in the correlation data (*r*), standardized coefficients (β), or path coefficients (γ), enhancing the reliability of our findings. These findings provide an important empirical basis for further investigation into the role of physical activity in promoting psychological resilience among young students and offer guidance for future research directions and methodologies.

The meta-analysis results indicate a positive impact of physical activity on the psychological resilience of young students, with a significant positive correlation observed between the two (average correlation coefficient *r* = 0.249, *p* < 0.001). This suggests that young students who actively participate in physical activities tend to exhibit higher levels of psychological resilience. This finding aligns with systematic reviews of cancer patients (CA) (Matzka et al., 2016) and individuals with disabilities (DP) (Jalilvand and Maleki, 2023), both of which report a positive correlation between physical activity and psychological resilience, particularly noting that higher levels of physical activity are associated with greater resilience.

Furthermore, a meta-analysis of multiple regression data revealed a significant positive correlation between increased physical activity and enhanced psychological resilience levels among young students, with an average standardized coefficient β of 0.270 (*p* < 0.001). As young students engage in higher levels of physical activity, their psychological resilience correspondingly strengthens. The results of this study underscore the pivotal role of physical activity in promoting the psychological resilience of young students. By participating in and persisting with physical exercise, young students can overcome various challenges, such as physical discomfort and adverse weather, thereby enhancing their psychological resilience. Several studies support this perspective. For instance, Li and Guo (2023) found that physical activity is a significant positive predictor of psychological resilience. Similarly, Guo and Liang (2023) employed a cross-lagged panel model to analyze the relationship between physical activity and resilience, concluding that there is a causal relationship between physical activity and the resilience of adolescents. Additionally, the literature suggests that improved psychological resilience promotes participation in physical activities. For example, Jefferies et al. (2019) mentioned that the relationship between psychological resilience and physical activity may be bidirectional, with individuals exhibiting greater resilience being more likely to engage in physical activities.

In the meta-analysis, structural equation model data further elucidated the association between the extent of young students' participation in physical activities and the enhancement of their psychological resilience (average path coefficient γ = 0.355, *p* < 0.001). This indicates that participation in physical activities has a positive effect on strengthening the psychological resilience of young students. This effect may be related to the positive impact of physical activity on health. For example, Cui and Zhang (2022) found that physical exercise helps reduce negative emotions in college students by enhancing their independence and intellect, thereby improving their psychological resilience in the face of unpleasant emotions. Similarly, San Román-Mata et al. (2020) demonstrated that active participation in health-beneficial physical activities can enhance psychological resilience and emotional management skills, reducing the incidence of psychological distress. These studies collectively highlight the positive role of physical activity in promoting the psychological resilience of young students and emphasize the importance of physical function and mental health in this process. Additionally, Dunston et al. (2022) found that individuals engaging in more vigorous physical activities scored higher in resilience and perseverance. This finding holds significant theoretical and practical implications, providing theoretical support for promoting physical activity among young students to improve their quality of life.

The relationship between physical activity and psychological resilience among young students is influenced by a variety of factors, including social support, exercise volume, school environment, quality of life, and interpersonal relationships. Among these, social support is a frequently cited determinant that plays a significant role in young students' engagement in physical activities and their psychological resilience. Liu et al. (2024) found that support from family, society, and teachers can enhance college students' motivation and willingness to participate in sports, which in turn facilitates their commitment to exercise, the initiation of exercise behaviors, and the cultivation of psychological resilience. Furthermore, Li and Guo (2023) demonstrated an interactive effect between physical activity levels and social support, which collectively promotes the psychological resilience of young students.

Exercise volume also has a notable impact on the relationship between physical activity and psychological resilience. Studies indicate that individuals engaged in high levels of physical activity exhibit greater resilience and a strong sense of personal determination (Chen et al., 2013; Chi et al., 2021; O'Neill, 2018; Wu et al., 2024). Dunston et al. (2022) noted that physical activity contributes to positive outcomes in psychological resilience, with resilience significantly increasing with the intensity of vigorous physical activity, a effect not observed with moderate-intensity exercise. The intensity of physical activity may be crucial, as vigorous activity has a stronger association with perseverance than moderate activity or walking, with sedentary behavior showing no correlation with resilience in college students (Dunston et al., 2022).

The school environment is highlighted as a persistent catalyst and source of motivation for enhancing adolescent wellbeing, directly promoting physical activity participation and the enhancement of psychological resilience (Li et al., 2024). Additionally, several studies (Dai and Menhas, 2020; Saqib et al., 2020; Fan et al., 2023) provide evidence for the positive role of quality of life factors in the relationship between regular physical activity and psychological resilience. Lastly, interpersonal relationships influence students' participation in physical exercise and their mental health. As Li et al. (2024) pointed out, a harmonious interpersonal environment satisfies students' strong need for a sense of belonging, thereby reinforcing their participation in physical exercise and contributing to their mental health. These findings underscore the importance of considering these multifaceted factors for a comprehensive understanding of the relationship between physical activity and psychological resilience among young students.

This study provides significant insights into the correlation between physical activity and psychological resilience among young students; however, it is subject to certain limitations. Firstly, the number of studies included is limited, and they predominantly come from countries such as China and the United States, which may constrain the generalizability of the research findings. Secondly, the research design is mainly cross-sectional, lacking longitudinal evidence, making it challenging to establish causal relationships. Additionally, there is insufficient consideration of potential influencing factors such as social support and environmental conditions. Furthermore, an overreliance on self-reported data may introduce bias. Future research should aim to enhance the geographical diversity of the sample, employ longitudinal study designs, and comprehensively consider all potential influencing factors. Moreover, it should focus on developing effective intervention strategies. Simultaneously, a deeper exploration of the physiological and psychological mechanisms is warranted to strengthen the scientific validity and practical applicability of the research.

The results of this study indicate that physical activity plays a significant role in promoting the mental health resilience of adolescents. To this end, schools should enrich extracurricular activities, offer a variety of sports options, improve sports facilities, create a favorable environment for physical activity, and implement mental health education. This will help students understand the importance of mental health, master psychological coping skills, and enhance their self-confidence and ability to deal with stress through sports psychology training. At the family level, parents should encourage their children to participate in sports, accompany them in physical exercises, foster an active family sports culture, and lead by example, engaging in sports themselves to set a positive precedent for their children. Moreover, schools and families need to collaborate to provide proactive social support for students, encouraging their participation in sports and offering necessary assistance and support. They should also pay attention to students' mental health status, promptly identify and help them cope with stress and challenges, and guide them to alleviate pressure through physical activity. Researchers should further conduct studies on sports interventions, exploring the mechanisms by which different types of exercise affect the mental health and resilience of adolescents. Customized intervention plans should be developed based on individual student characteristics, and the intrinsic connections between exercise, mental health, and resilience should be investigated in depth. For instance, examining the regulatory effects of exercise on brain neurotransmitters and the immune system will provide a more comprehensive understanding of the scientific mechanisms by which exercise promotes mental health and resilience. Through the collaborative efforts across these multiple levels, the mental health and resilience of young students can be effectively enhanced, equipping them to better cope with life's challenges and improving their overall quality of life.

## Conclusion

This study validates a significant positive correlation between the psychological resilience of young students and their physical activity, with physical activity exerting a promotional effect on resilience and psychological resilience in turn facilitating participation in physical activities. These findings provide theoretical support for enhancing the psychological resilience and quality of life of young students. Future research should investigate the specific roles of various influencing factors and develop targeted interventions aimed at motivating young students to engage more actively in physical activities, thereby improving their psychological resilience.

## Data Availability

The original contributions presented in the study are included in the article/supplementary material, further inquiries can be directed to the corresponding authors.
